# Screening of Almond Germplasm for Bioactive-Rich Skin Recovery and Application of Liquid Nitrogen Peeling

**DOI:** 10.3390/foods15101668

**Published:** 2026-05-11

**Authors:** Fabiola Pesce, Giovanni Spagna, Lucia Parafati, Aldo Todaro, Gaetano Distefano, Leonardo Luca, Rosa Palmeri

**Affiliations:** Di3A, Dipartimento di Agricoltura, Alimentazione e Ambiente, University of Catania, Via S. Sofia 100, 95123 Catania, Italy; fabiola.pesce@phd.unict.it (F.P.); aldo.todaro@unict.it (A.T.); gaetano.distefano@unict.it (G.D.); leonardo.luca@unict.it (L.L.); rosa.palmeri@unict.it (R.P.)

**Keywords:** almond skin, liquid nitrogen peeling, polyphenols, antioxidant activity, by-products valorization, *Prunus dulcis*

## Abstract

Almond skins represent an underexploited by-product of the almond processing chain, despite being a rich source of bioactive compounds with potential applications as a functional food ingredient. However, the Traditional Blanching Peeling (TBP) process, which involves the use of hot water, causes the leaching and degradation of bioactive compounds, reducing the functional value of the recovered skins. In this study, liquid nitrogen peeling (LNP) was investigated as an alternative approach to enhance the recovery of bioactive-rich almond skins. To assess the variability of bioactive compounds in different cultivars, a total of 106 almond genotypes from a germplasm collection were screened for skin yield, total polyphenol content, and antioxidant activity. Principal component analysis (PCA) was applied to select a representative subset of cultivars capturing the variability of the germplasm in terms of bioactive composition and technological traits. The skins obtained using LNP exhibited higher polyphenol content and antioxidant activity compared to those obtained using conventional peeling. Despite variability among samples, in some cultivars such as ‘Universo’, TPC increased from 570.58 mg GAE/100 g after TBP to 1588.05 mg GAE/100 g after LNP, corresponding to an increase of approximately 178% (*p* < 0.05). Moreover, in all selected cultivars, kernel color (CIELab*) was not significantly affected by LNP, confirming the preservation of kernel quality. These results highlight the importance of cultivar selection and support LNP as a promising approach for the valorization of almond skins as functional by-products.

## 1. Introduction

Nowadays, the transition towards more sustainable food systems has become necessary for both environmental and economic reasons. In this context, the food industry is increasingly focusing on the valorization of agro-industrial by-products as a key strategy to reduce waste and promote circular economy approaches within the food chain [1,2].

Several research studies have explored the reuse of a wide range of by-products for the development of novel food ingredients, which can be employed as emulsifiers, stabilizers, or sources of proteins and other nutrients [3,4]. Among these, particular attention has been given to by-products rich in bioactive compounds, due to their potential application as functional ingredients for the fortification of food formulations. Moreover, this compound may exhibit antimicrobial activity, contributing to the extension of food product shelf life [5,6].

Almond (*Prunus dulcis* (Mill.) D.A. Webb) is one of the most widely cultivated nut crops worldwide. Its germplasm collection is a strategic resource to conserve and to explore phenotypic and genetic variability to characterize fruit quality and enhanced stress resistance [7]. This diversity broadens the genetic base of elite material, increases its adaptive potential, and explores specific quality traits through the screening of neglected almond cultivar collections [8]. In particular, almond kernels are extensively used as an ingredient in bakery and confectionery products or consumed in their entirety, either fresh or following roasting processes. However, almond processing involves a peeling step that requires significant water and energy inputs and generates large amounts of by-products, including hulls, shells, skins, and blanching water. Conventional hot-water peeling, widely applied at the industrial level, promotes the leaching of polyphenols into the peeling water (blanching water) and induces kernel oxidative degradation [9], thereby reducing the functional quality of the recovered skins [10].

The main by-products of this process are therefore blanched skins and blanching water, both of which have been investigated for potential reuse. Indeed, several studies have focused on the valorization of both blanched skins and blanching water as a valuable source of bioactive compounds [11,12]. Blanched skins, thanks to the possibility of being simply used as a flour, represent the main investigated almond by-products, especially as an ingredient in the bakery industry, to improve their functional properties [11,13,14,15].

Almond skins are particularly rich in phenolic compounds, including flavonoids and phenolic acids, which are responsible for their strong antioxidant activity [16]. Moreover, almond skin extracts have been reported to exhibit antimicrobial, anti-inflammatory, and antiviral properties, highlighting their potential as functional ingredients and nutraceuticals. In addition, almond skins have shown prebiotic effects on the intestinal microbiota [17]. Due to its intrinsic properties, such as instability and the need for large storage volumes, blanching water is less suitable for direct industrial reuse compared to the solid fraction. Nevertheless, the amount of bioactive compounds degraded or lost in the peeling water can vary significantly depending on the almond cultivar [16,18].

As already highlighted by studies on almond germplasm variability [19,20], significant differences in polyphenol content and antioxidant activity can be observed among cultivars, suggesting that the functional potential of almond by-products may vary considerably depending on genotype and its response to processing conditions. In particular, cultivar-dependent differences may influence both the degradation and the release of polyphenols during hot-water peeling.

In this context, innovative peeling technologies may represent a promising approach to reduce the environmental impact associated with high water and energy consumption, while enhancing the recovery of high-quality almond skins. Cryo-peeling processes have been investigated in other nut matrices, such as walnuts and pistachios [21,22]. In this latest matrix, liquid nitrogen (LN_2_) treatments have been shown to reduce thermal degradation and phenolic losses, thereby preserving the bioactive profile of the skins while maintaining kernel integrity [22]. However, these studies mainly focused on process feasibility or quality preservation in limited sample sets, and the application of cryogenic peeling to almond germplasm has not yet been explored. To the best of our knowledge, the present study represents the first application of liquid nitrogen peeling to a large-scale almond germplasm collection (n = 106), allowing the evaluation of cultivar-dependent responses in terms of bioactive compound recovery. Compared to conventional blanching, liquid nitrogen peeling eliminates hot water usage and avoids the subsequent drying step, both of which are energy-intensive, while also minimizing thermal degradation and the leaching of polyphenols [22]. This study identifies genotypes best suited to this treatment in terms of preserving and recovering bioactive compounds that are otherwise lost in peeling water during conventional processing. The optimization of the peeling process, particularly with respect to maximizing the yield of intact kernels, was beyond the scope of the present research, which focused on cultivar-dependent responses in terms of bioactive compound recovery.

Therefore, the aims of the present study were to (a) perform a large-scale screening by evaluating skin yield, total polyphenol content, and antioxidant activity on a germplasm collection encompassing 106 almond cultivars; (b) apply a multivariate approach (PCA) to ensure a balanced selection of genotypes with contrasting profiles; (c) compare almond skins obtained by liquid nitrogen peeling and conventional hot-water peeling in terms of polyphenol content and antioxidant activity and evaluate phenolic losses during peeling through the analysis of peeling water; (d) assess kernel quality after LN_2_ treatment through color analysis (CIELab*) in order to verify the applicability of the process and exclude detrimental effects on the edible fraction.

## 2. Materials and Methods

### 2.1. Plant Material

A total of 106 almond cultivars were analyzed in this study. The samples belonged to the ex situ germplasm collection preserved at the Agricultural Experimental Farm of the University of Catania (37°24′37.8″ N, 15°03′26.6″ E) and included cultivars of predominantly Sicilian origin, as well as Apulian and International varieties, thus representing a broad range of phenotypic and genetic diversity.

### 2.2. Application of Traditional Blanching Peeling (TBP) to 106 Almond Cultivars and Evaluation of Skin Yield

Traditional Blanching Peeling (TBP) was performed by progressively immersing 20 g of almonds in 35 mL of hot water (90 ± 1 °C) for 5 min, in order to ensure uniform heat transfer and reproducibility under controlled laboratory conditions and in order to avoid excessive dilution of the blanching water. Following peeling, kernels and skins were manually separated and subsequently dried at 40 °C for 48 h in a ventilated oven (Thermo Scientific, Heratherm™ oven, Milano, Italy) to remove residual moisture. The peeling water obtained after treatment was filtered through Whatman No. 1 filter paper and collected for subsequent analyses.

Skin yield evaluation was expressed as the ratio between the weight of skins and the weight of the peeled almond kernels, according to the following equation:

Skin yield % = (Skin weight)/(Kernel weight) × 100

where “Skin weight” is the weight of the recovered skin and “Kernel weight” is the weight of the corresponding peeled almonds. This parameter was used to evaluate differences among cultivars and to assess the effect of the peeling process on skin recovery. Skin yield values obtained for each cultivar were further used for the principal component analysis (PCA) described in Section 2.3.

#### Evaluation of Total Polyphenol Content and Antioxidant Activity in By-Products Obtained from Traditional Blanching Peeling (TBP)

**Figure 1:**
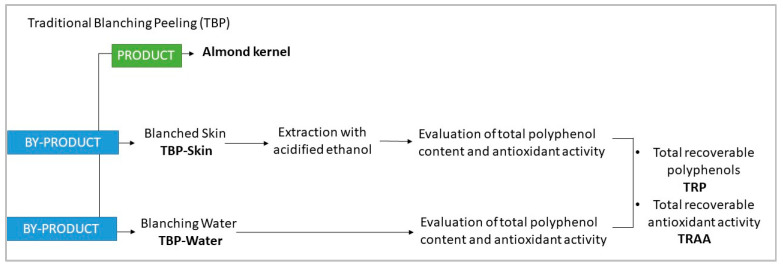
Schematic representation of by-product generation and bioactive compound distribution during Traditional Blanching Peeling (TBP).

Traditional Blanching Peeling (TBP), performed as described above, generated two by-products: blanched skins (TBP-Skin) and blanching water (TBP-Water). As schematized in Figure 1, both fractions were analyzed for total polyphenol content and antioxidant activity to quantify, across the 106 almond cultivars, the loss of bioactive compounds from the skins into the blanching water.

In order to maximize polyphenol recovery, the extraction of polyphenols from TBP-Skin samples was preliminarily optimized using a reference cultivar (Fascionello), selected due to its high polyphenol content in the skin fraction. This approach was adopted to minimize potential limitations related to analytical sensitivity and to ensure that differences among extraction conditions were not masked by low initial levels of bioactive compounds. Different solvent systems were tested during the optimization phase, including distilled water; water acidified with 1% HCl (0.1 N); water at 40 °C; water at 40 °C acidified with 1% HCl (0.1 N); methanol; methanol acidified with 1% HCl (0.1 N); ethanol; ethanol acidified with 1% HCl (0.1 N); isopropanol acidified with 1% HCl (0.1 N); and ethyl acetate (Figure S1, Supplementary Material). Among the tested solvents, ethanol acidified with 1% HCl (0.1 N) resulted in the highest extraction efficiency and was therefore selected for further extraction procedure.

Briefly, 0.5 g of almond skins were mixed with 15 mL of ethanol acidified with 1% HCl (0.1 N). The extraction was carried out for 1 h at room temperature under continuous agitation. After extraction, the suspensions were filtered through Whatman filter paper, and the resulting filtrates were collected and used for the determination of total polyphenol content and antioxidant activity.

Total polyphenol content (TPC) of peeling waters and almond skin extracts was determined using the Folin–Ciocalteu colorimetric assay, according to [23]. Briefly, 250 µL of each sample extract was reacted with the Folin–Ciocalteu reagent (Carlo Erba Reagents, Milan, Italy), followed by the addition of 2.5 mL of 20% (*w*/*v*) sodium carbonate solution. The reaction mixture was incubated for 1 h in the dark at room temperature to allow complete color development. Absorbance was measured at 725 nm using a PerkinElmer Lambda 25 UV/VIS spectrometer (PerkinElmer Inc., Waltham, MA, USA). Polyphenol content was quantified using a calibration curve prepared with gallic acid as the standard and expressed as mg gallic acid equivalents per 100 g of almonds (mg GAE/100 g). The Total Recoverable Polyphenols (TRPs) were calculated as the sum of polyphenols present in the blanching water and those remaining in the exhausted skins, in order to account for the overall distribution of polyphenols after peeling (Figure 1).

Antioxidant activity (AA) of peeling waters and almond skin extracts was determined by the DPPH radical scavenging assay according to [24]. Briefly, 50 μL of sample extract was added to 3 mL of 100 μM DPPH solution. After 1 h of incubation in the dark at room temperature, absorbance was measured at 515 nm. AA was expressed as µg of Trolox Equivalents (TE)/100 g of almonds, using a calibration curve prepared with Trolox as reference standard over the concentration range of 0–750 µg/L.

The overall antioxidant activity, defined as Total Recoverable Antioxidant Activity (TRAA) (Figure 1), was calculated by summing the antioxidant activity measured in the blanching water and that determined in the exhausted skins.

Data related to TRP and TRAA were used for multivariate statistical analysis, in order to ensure a consistent quantitative input for principal component analysis (PCA). The resulting total polyphenol values, expressed as mg GAE/100 g of almonds, were used for principal component analysis (PCA).

### 2.3. Selection of Almond Cultivars and Application of Liquid Nitrogen Peeling

Cultivars selected for liquid nitrogen peeling (LNP) were identified using principal component analysis (PCA) in order to select a representative subset capturing the variability of the germplasm in terms of phenolic composition, antioxidant activity, and skin yield.

PCA was performed on standardized variables (z-scores), including Total Recoverable Polyphenols (TRPs) (expressed as mg GAE/100 g of almonds), Total Recoverable Antioxidant Activity (TRAA) (Figure 1) (expressed as µg TE/100 g of almonds), and skin yield (%) (see Section 2.2).

Liquid nitrogen peeling (LNP) of almond kernels was carried out by exposing samples to nitrogen vapors generated by the evaporation of liquid nitrogen (LN_2_), with minor modifications to the procedure reported by [22]. In detail, kernels were placed on a perforated grid at a controlled distance (approximately 1–3 cm) from the LN_2_ surface to ensure uniform exposure to cryogenic vapors for 10–15 min. Exposure time and distance from the nitrogen surface were adjusted depending on the cultivar to optimize skin detachment while maintaining kernel integrity. Following treatment, the skins were manually removed as in conventional peeling. No additional drying was required due to the absence of water during the process.

#### Bioactive Compounds of Skin and Color Parameters of Kernel Subjected to Traditional Blanching Peeling (TBP) and Liquid Nitrogen Peeling (LNP)

After PCA, the selected almond cultivars, were subjected to liquid nitrogen peeling (LNP) according to the method described by [22] . Briefly, almonds were exposed to liquid nitrogen vapors, inducing rapid thermal stress at the skin-kernel interface, and subsequently manually peeled. The detached skin of each sample was collected and used for further analyses.

Almond skins obtained after LNP were subjected to two consecutive extraction steps to ensure comprehensive recovery of polyphenols. Unlike traditional peeling, LNP treatment does not involve water contact, thus preserving both ethanol-soluble and highly polar polyphenol fractions within the skin matrix. The optimized extraction method involved two consecutive steps: a first extraction was performed using acidified ethanol (1% HCl) as described in Section 2.2, followed by a second extraction with distilled water at room temperature under agitation to recover residual water-soluble compounds. Both ethanolic and aqueous extracts were collected separately and used for the determination of total polyphenol content and antioxidant activity of skins obtained from LNP (LNP-Skin), as described in Section 2.2.

To exclude potential side effects of LNP on the almond kernel, color was evaluated as a key parameter closely related to lipid oxidation. The color of almond kernels obtained after TBP and LNP was measured by instrumental colorimetric analysis. Measurements were performed using a portable colorimeter (Konica Minolta CM-2500d, Bremen, Germany) equipped with illuminant D65. For each treatment, kernel samples were placed in the appropriate measurement accessory (CR-A50) and color parameters were recorded according to the CIELab* color space. The following coordinates were measured: lightness (L*), redness (a*), and yellowness (b*). Moreover, the derived color parameters Chroma (C*) and hue angle (h°) were calculated from the CIELab* coordinates (a*, b*). Chroma (C*) represents color saturation, while hue angle (h°) describes the tonal attribute of the color. The parameters were calculated according to the following equation:

$$C={\left({a*}^{2}+b{*}^{2}\right)}^{1/2}h{}^{\circ}={tan}^{-1}\left(\frac{b*}{a*}\right)$$

### 2.4. Statistical Analysis

Data were expressed as mean ± standard deviation of at least three independent replicates. Microsoft Excel (2013) was used only for data organization and preliminary calculations. All statistical analyses were performed using Minitab™ software (version 20.0, Minitab Inc., State College, PA, USA). One-way analysis of variance (ANOVA), followed by Fisher’s least significant difference (LSD) test (*p* < 0.05), was applied to evaluate differences among extraction solvent systems during the optimization step. For the comparison between peeling methods (Traditional Blanching Peeling, TBP, and liquid nitrogen peeling, LNP), statistical analyses were performed on the overall dataset for each variable. Normality and homogeneity of variance were assessed using the Shapiro–Wilk and Levene tests, respectively. Depending on the outcome of these tests, either Student’s t-test (equal variances) or Welch’s t-test (unequal variances) was applied (*p* < 0.05). Values reported for individual cultivars, as reported in Section 3.3.2, are descriptive and were not subjected to separate hypothesis testing at the cultivar level. Principal component analysis (PCA) was performed using Minitab™ (version 20.0) to explore the multivariate structure of the dataset and the distribution of cultivars.

## 3. Results and Discussion

### 3.1. Skin Yield

**Figure 2:**
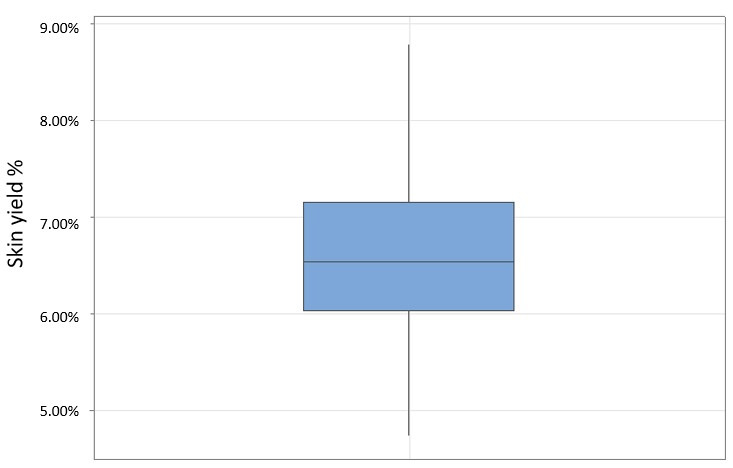
Boxplot distribution of skin yield (%) across the 106 almond cultivars. The box represents the interquartile range (IQR), the central line indicates the median, and whiskers represent the minimum and maximum values.

Skin yield (%), from highest to lowest value, of each almond sample is reported in Table 1. The observed range extended from 8.78%. to 4.74%, with the highest skin yields observed in ‘Don Pitrino’ (8.78%), ‘Enna 2’ (8.73%), ‘Bennici’ (8.67%), ‘Miuzza’ (8.60%), and ‘Buscarina’ (8.58%). The lowest value was recorded for ‘Cavalera Di Naro’ (4.74%), followed by ‘Muddisa Tonda’ (4.83%) and ‘Fascionello’ (4.87%). Nevertheless, most of the accessions encompassing the population under study displayed skin value ranging from 6.0% to 7.5. Intermediate values were recorded for cultivars such as ‘Scummissa’ (6.75%) and ‘Sancisuca (Soat Licata)’ (6.32%). The distribution of skin yield across the 106 cultivars is summarized in Figure 2, which highlights the relatively narrow variability of this parameter, with most values clustered around the median. The interquartile range is limited, indicating that the majority of cultivars fall within a similar yield interval. Moreover, the absence of extreme outliers suggests a relatively homogeneous distribution of skin proportion among the analyzed genotypes.

These findings reveal substantial genotype-dependent variation in skin proportion and yield among the cultivars analyzed, with values ranging from 4.74% to 8.78%, reflecting differences in kernel structural traits. Although skin proportion is not commonly reported as a primary agronomic descriptor, it represents a technologically relevant parameter in almond processing, as the tegument constitutes the main fraction removed during blanching and peeling operations [25]. Differences in skin yield may therefore influence mass balance and by-product generation across cultivars [26]. Morphological variability in kernel components has been documented in Mediterranean almond germplasm, highlighting substantial genetic diversity in structural traits [27]. The variability observed in the present study is consistent with this genotype-driven heterogeneity. Given that the almond tegument is known to contain the highest concentration of polyphenols [28,29], differences in skin proportion among cultivars may also contribute to compositional variability, which is discussed in detail in the following section.

#### 3.1.1. Polyphenol Content in Almond By-Product Obtained Through Traditional Blanching Peeling

As displayed in Figure 1, the traditional blanching process (TBP) generates two by-products that can be exploited as a source of bioactive compounds. Table 2 reports the total polyphenol content in blanched skins (TBP-Skin) and blanching water (TBP-Water) as well as the Total Recoverable Polyphenols (TRPs), calculated as the sum of polyphenols present in the blanching water and those remaining in the exhausted skins (Figure 1).

Samples are listed in Table 2 in descending order according to their TRP value. Moreover, based on TRP value, almond cultivars were classified into three groups: high (TPC > 400 mg GAE/100 g), medium (TPC between 200 and 400 mg GAE/100 g), and low (TPC < 200 mg GAE/100 g). A wide variability among cultivars was observed, ranging from 834.49 mg GAE/100 g in ‘Pizzutella’ to 52.77 mg GAE/100 g in ‘Palma’, with most cultivars showing a medium polyphenol content with values ranging between 150 and 400 mg GAE/100 g.

This variability is consistent with previous studies reporting that the phenolic composition of almonds is strongly influenced by cultivar, as well as by environmental and agronomic factors [29].

A particularly relevant aspect emerging from the results concerns the distribution of polyphenols among the analyzed fractions. In most cultivars, the majority of polyphenols was detected in the blanching water, whereas measurable amounts remained in the skin fraction only in a limited number of accessions (such as for ‘Tuono’ and ‘Fascionello’ cultivars, Table 2). This behavior can be explained by the combined effect of thermal and mass transfer phenomena occurring during blanching. High temperatures promote the disruption of cell structures, increasing membrane permeability and facilitating the release of intracellular polyphenols [10,29]. At the same time, the aqueous environment enhances the solubility of these compounds, promoting their diffusion from the skin matrix into the surrounding medium [10].

Although almond skins are widely recognized as the primary reservoir of phenolic compounds in almonds [29], the results show that blanching leads to a substantial loss of polyphenols in the processing water, thereby reducing the amount of bioactive compounds retained in the solid fraction. Similar observations were reported by [30], where blanch water contained appreciable amounts of polyphenols following thermal treatment.

In light of these findings, alternative peeling strategies that avoid hot water contact and limit thermal stress, such as liquid nitrogen peeling, may represent a promising approach to limit the loss of polyphenols and preserve the content of bioactive molecules in almond skins, thereby maintaining their functional value.

#### 3.1.2. Antioxidant Activity in Almond By-Product Obtained Through Traditional Blanching Peeling

Table 3 reports the antioxidant activity of blanched skins (TBP-Skin) and blanching water (TBP-Water) and the Total Recoverable Antioxidant Activity (TRAA), calculated as the sum of the antioxidant activity measured in the blanching water and in the exhausted skins (as schematized in Figure 1). Samples are listed in Table 3 in descending order according to their TRAA value. Additionally, based on TRAA value, almond cultivars were classified into three groups: High (>2500 µg TE/100 g), Medium (2000–2500 µg TE/100 g), and Low (<2000 µg TE/100 g).

The TRAA of almond cultivars ranged from 3130.25 µg TE/100 g in ‘Nivera Manza’ to 1651.77 µg TE/100 g in the cultivar ‘Calamonaci’, with most cultivars showing values between 1800 and 2400 µg TE/100 g. Such variability is consistent with previous studies reporting that antioxidant capacity in almonds is strongly influenced by cultivar and by the phenolic composition of the kernels and skin [25,29].

Similarly to what was observed for TPC (Table 2), the highest antioxidant activity was generally detected in the peeling water fraction, whereas the skin fraction contributed only a limited portion of the TRAA in most cultivars (Table 3). These findings are consistent with the well-established role of polyphenols as major contributors to the antioxidant capacity of almond tissues, particularly in the skin fraction [29,31,32]. During the conventional blanching process, however, the disruption of cellular structures and the increased solubility of phenolic molecules in water can promote their diffusion into the blanching medium [10]. As a consequence, antioxidant compounds originally associated with almond skin may be partially transferred to the peeling water, leading to a redistribution of antioxidant activity between the solid and liquid fractions.

Similar behavior has been described in previous studies investigating almond processing by-products, where blanching water was found to contain appreciable amounts of antioxidant compounds released from almond skins during thermal treatment [30]. Due to its bioactive compound content, blanching water has also been proposed for use in food formulations [11].

Overall, these findings confirm that a significant fraction of antioxidant compounds may be lost into the processing water during conventional blanching, potentially reducing the antioxidant potential retained in the skin fraction. From a technological perspective, this aspect highlights the importance of developing alternative peeling strategies capable of preserving phenolic compounds associated with almond skin [33].

### 3.2. Selection of Representative Almond Cultivars for Liquid Nitrogen Peeling (LNP)

**Figure 3:**
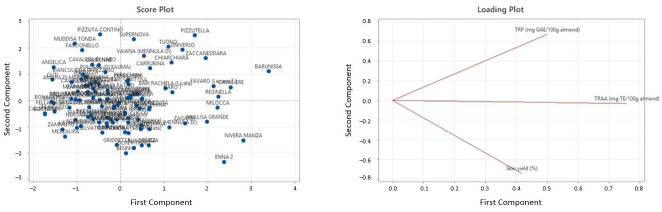
PCA score plot and loading plot of almond cultivars based on Total Recoverable Polyphenols (TRPs), Total Recoverable Antioxidant Activity (TRAA), and skin yield (%).

The cultivars exhibiting the highest technological potential and the highest bioactive compound concentration in the skin were identified based on a principal component analysis (PCA) performed using Total Recoverable Polyphenols (TRPs), Total Recoverable Antioxidant Activity (TRAA), and skin yield (%) values (Figure 3).

The first two principal components (PC1 and PC2) explained 36.8% and 34.6% of the total variance, respectively, accounting for a cumulative variance of 71.4%, thus providing a reliable representation of the variability within the dataset.

The selected cultivars were distributed across different regions of the PCA score plot in order to capture the variability of the almond germplasm in terms of polyphenols, antioxidant activity, and skin yield. In particular, PC1 was mainly associated with TRAA, whereas PC2 was primarily influenced by TRP and skin yield.

Based on their position in the score plot, cultivars were selected from all four quadrants to obtain a representative subset of the germplasm (Table 4). Cultivars located in quadrant I were considered the most promising, as they combined high values of bioactive compounds and favorable technological traits. Quadrant II included cultivars characterized by relatively high polyphenol content with a lower contribution of antioxidant activity and/or yield. Quadrant IV comprised genotypes with higher antioxidant activity and/or skin yield but lower PC2 scores, whereas quadrant III included cultivars with lower bioactive content, retained as reference samples.

### 3.3. Effect of Liquid Nitrogen Peeling (LNP) on Almond Skins and Kernel Quality Compared to Traditional Blanching Peeling (TBP)

#### 3.3.1. Total Polyphenol Content and Antioxidant Activity of Skin

**Figure 4:**
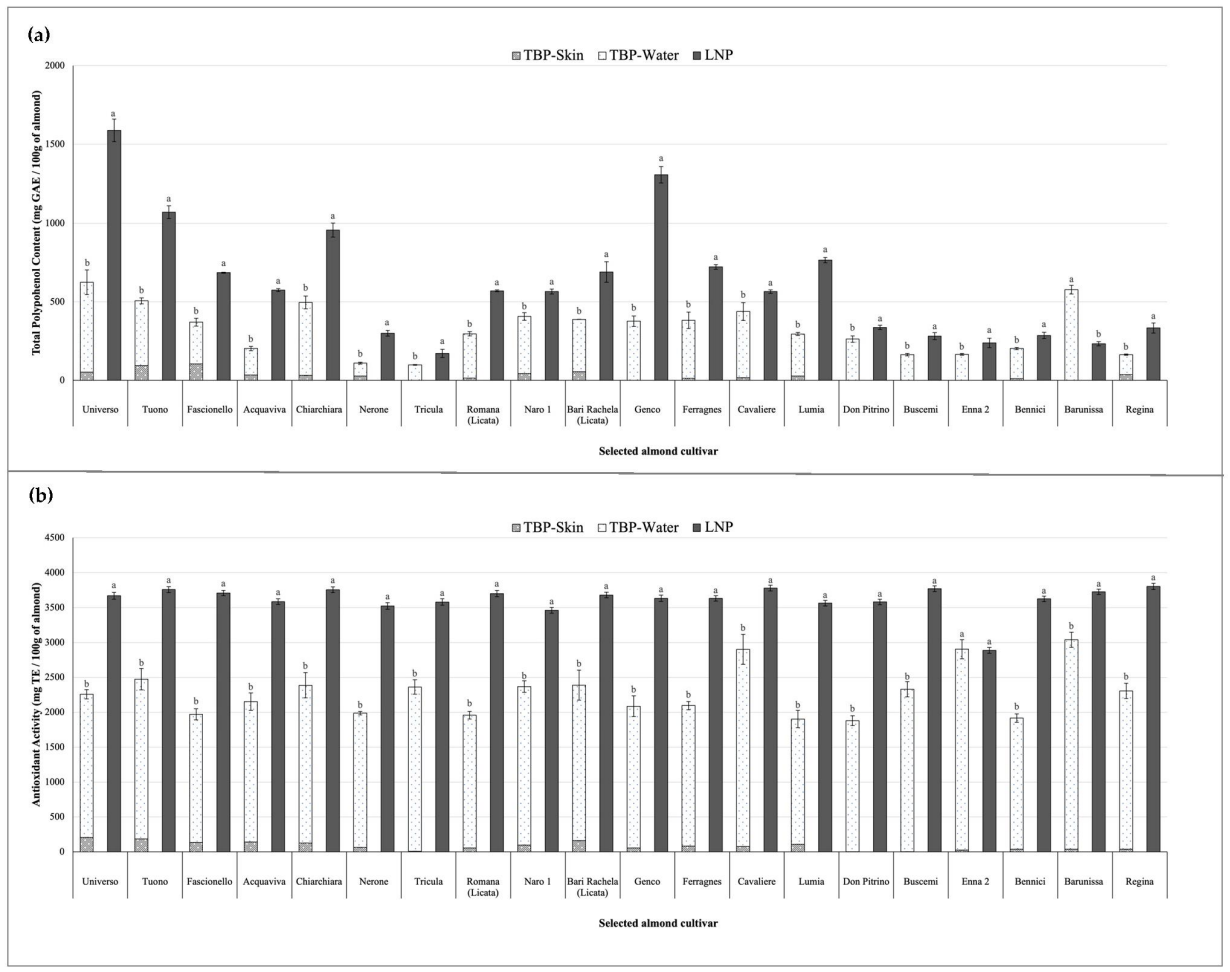
Comparison of total polyphenol content (**a**) and antioxidant activity (**b**) in almond skins and blanching water obtained by Traditional Blanching Peeling (TBP) and liquid nitrogen peeling (LNP). Results are expressed as mg GAE/100 g (**a**) and µg TE/100 g (**b**) of almonds (mean ± standard deviation). Values are expressed as mean ± standard deviation (n = 3). Different letters indicate statistically significant differences (*p* < 0.05) according to the applied test (ANOVA with Fisher’s LSD or Mann–Whitney test, as appropriate).

Figure 4a,b showing the comparison between TBP and LNP revealed substantial differences in the retention of bioactive compounds in almond skin. While TBP resulted in a significant loss of polyphenols due to their migration into the peeling water, LNP allowed a markedly higher retention of these bioactive molecules in the peeled almonds (Figure 4a).

Across all analyzed cultivars, total polyphenol content (TPC) was significantly higher after LNP compared to TBP (*p* < 0.05), although the magnitude of the increase was cultivar-dependent. In the analyzed cultivars, TPC values measured after LNP ranged from 171.76 mg GAE/100 g in ‘Tricula’ to 1588.05 mg GAE/100 g in ‘Universo’, showing a clear increase compared with the total polyphenol content previously measured after TBP. For instance, in the cultivar ‘Universo’, TPC increased from 570.58 mg GAE/100 g after TBP to 1588.05 mg GAE/100 g after LNP, corresponding to a significant (*p* < 0.05) increase of approximately 178%. A similar behavior was observed in ‘Genco’, where TPC increased from 376.94 mg GAE/100 g to 1306.05 mg GAE/100 g, represent a significantly (*p* < 0.05) increase of about 247%, and in ‘Tuono’, where polyphenol content significantly (*p* < 0.05) increased from 475.48 mg GAE/100 g to 1070.93 mg GAE/100 g (+125%, *p* < 0.05). Even cultivars characterized by relatively low polyphenol levels after TBP showed a marked increase following cryogenic peeling. For example, ‘Nerone’ significantly (*p* < 0.05) increased from 109.64 mg GAE/100 g to 300.12 mg GAE/100 g (+174%), while ‘Tricula’ significantly (*p* < 0.05) increased from 99.02 mg GAE/100 g to 171.76 mg GAE/100 g (+73%). Only limited differences were observed in some cultivars, such as ‘Cavaliere’, where TPC increased from 438.74 mg GAE/100 g after TBP to 565.81 mg GAE/100 g after LNP (+29%, *p* < 0.05) (Figure 4a).

These results highlight the strong influence of the peeling technology on the retention of polyphenols. During TBP, the combination of heat and water promotes the diffusion of water-soluble polyphenols from almond skin into the surrounding medium, leading to substantial losses of these bioactive molecules in the blanching water [10,16,30]. Since almond skin represents the main reservoir of polyphenols in the kernel, the removal of the tegument during processing can significantly influence the overall phenolic composition of the product [29]. In addition to leaching phenomena, thermal processing may also contribute to partial degradation or oxidation of phenolic compounds, further reducing the concentration of antioxidant compounds retained in the edible fraction.

Conversely, LNP removes the skin through rapid thermal shock without water contact, thereby limiting the diffusion of polyphenols and reducing thermal degradation phenomena. As a consequence, LNP allows a markedly higher preservation of polyphenol compared with traditional hot-water peeling. Similar preservation effects associated with cryogenic peeling processes have been previously reported in studies investigating alternative peeling technologies for nuts [22]. These findings demonstrate that the peeling method plays a crucial role in determining the final phenolic composition of almonds and that the use of liquid nitrogen may represent an effective strategy to preserve the bioactive potential of the product.

The comparison between TBP and LNP also revealed marked differences in antioxidant activity among the analyzed cultivars (Figure 4b). In general, samples obtained after LNP showed higher antioxidant activity compared with those obtained through conventional blanching. For example, the cultivar ‘Universo’ exhibited an antioxidant activity of 3668.87 µg TE/100 g after LNP, compared with a total value of 2258.41 µg TE/100 g measured after TBP, corresponding to a significant (*p* < 0.05) increase of approximately 62%. A similar trend was observed in ‘Tuono’, where antioxidant activity significantly (*p* < 0.05) increased from 2473.91 mg TE/100 g to 3757.48 µg TE/100 g (+52%), and in ‘Fascionello’, where values significantly (*p* < 0.05) improved from 1969.46 µg TE/100 g to 3708.51 mg TE/100 g, corresponding to an increase of about 88% (*p* < 0.05). Substantial increases were also observed in cultivars with moderate antioxidant activity after TBP. For instance, ‘Nerone’ increased from 1986.84 µg TE/100 g to 3521.96 µg TE/100 g (+77%, *p* < 0.05), while ‘Buscemi’ showed a significant (*p* < 0.05) increase from 2330.54 µg TE/100 g to 3770.31 µg TE/100 g (+62%). Even in cultivars where the increase was less pronounced, such as ‘Enna 2’, antioxidant activity remained comparable between treatments, with values of 2902.93 µg TE/100 g after TBP and 2886.71 µg TE/100 g after LNP (Figure 4b).

The observed trend closely follows the variation previously described for total polyphenol content, confirming the major contribution of polyphenols to the antioxidant potential of almonds (almond polyphenols: methods of analysis, contribution to food quality, and health promotion). As discussed above, conventional blanching promotes the migration of antioxidant compounds into the peeling water, reducing the amount retained in the edible fraction. In contrast, the absence of water contact during cryogenic peeling limits the diffusion of polyphenols and allows their retention within the almond tissues, resulting in higher antioxidant activity values, as already evaluated in other food matrices [22].

Overall, these findings confirm that the peeling technology plays a key role not only in determining phenolic composition but also in preserving the antioxidant potential of almonds.

#### 3.3.2. Kernel Color Parameters

The colorimetric parameters of almond kernels obtained by TBP and LNP are reported in Table 5. All color parameters showed normal distributions, while variance homogeneity varied across variables. Accordingly, statistical comparisons between TBP and LNP were performed on the overall dataset for each color parameter using the appropriate two-sample t-test. Significant differences were observed for L*, a*, and h°, whereas b* and C* did not differ significantly between treatments (*p* > 0.05).

Regarding lightness (L), values obtained after TBP ranged from 73.06 ± 0.27 (‘Cavaliere’) to 80.51 ± 0.45 (‘Bari Rachela (Licata)’), whereas samples obtained by LNP showed a slightly higher range, varying from 75.64 ± 0.53 (‘Cavaliere’) to 83.24 ± 0.18 (‘Buscemi’). Although the magnitude of the difference varied among cultivars, the overall statistical analysis confirmed significantly higher L values in LNP-treated samples (*p* < 0.05).

The a* parameter, which describes the red–green color component, showed marked variability among cultivars. The lowest values were observed in the cultivar ‘Regina’ (0.96 ± 0.11 for TBP), while the highest values were recorded in ‘Fascionello’ (7.08 ± 0.48 for TBP). Despite this variability, the global comparison between treatments showed a significant overall reduction in a values in LNP samples (*p* < 0.05). Conversely, in some cultivars such as ‘Regina’, the values remained very similar between treatments.

For the b* parameter, representing the yellow color component, no statistically significant differences were detected between TBP and LNP in the overall analysis (*p* > 0.05). Although TBP samples often showed slightly higher mean values, these variations were small and not statistically meaningful. For instance, in the cultivar ‘Romana (Licata)’, the b* value was 24.24 ± 0.34 after TBP and 22.91 ± 0.26 after LNP, but this difference was not significant. A similar pattern was observed in ‘Fascionello’, where b* decreased from 23.06 ± 0.46 to 21.48 ± 0.27, again without statistical significance.

The variations observed in a* and b* were also reflected in the derived parameters C* (Chroma) and h° (hue angle). Chroma showed only minor numerical differences between treatments, which were not statistically significant (*p* > 0.05). In contrast, the hue angle (h°) differed significantly between TBP and LNP in the overall analysis (*p* < 0.05), indicating a shift in overall color tone associated with the peeling method.

When comparing the L* values obtained with the two peeling methods, almonds subjected to LNP generally showed higher lightness values than those obtained through TBP. This difference may be related to the thermal treatment applied during the blanching process. Blanching followed by drying may promote Maillard reactions and oxidative phenomena, which can lead to partial browning of the kernels and consequently lower L* values [34]. In contrast, the use of liquid nitrogen allows the removal of the skin without exposing the kernels to high temperatures, thus reducing the risk of thermally induced color changes and helping preserve the natural appearance of the kernels. These results are consistent with previous studies showing that temperature and storage conditions play an important role in almond color stability. Effect of storage atmosphere and temperature on the oxidative stability of almond kernels during long-term storage reported that peeled almonds stored at lower temperatures maintained higher L* values over time compared with samples stored at room temperature, confirming that oxidative and browning reactions can progressively affect kernel color.

Thermal treatments are also known to influence other color coordinates. In the present study, although TBP samples tended to show slightly higher b* and C* values, these differences were not statistically significant (*p* > 0.05), indicating that the yellow component and color saturation were largely preserved regardless of the peeling method. Similar tendencies have been described in studies investigating the kinetics of color changes in almonds during roasting, where increases in processing temperature and treatment time resulted in progressive changes in color parameters [35,36]. In both studies, color degradation during thermal processing followed first-order reaction kinetics, increasing linearly with temperature and exposure time.

More recently, the study of Lipan et al. [9] reported that industrial processing steps such as shelling and blanching can significantly influence almond kernel color and integrity, further confirming that processing conditions play a key role in determining the final visual quality of almonds. Overall, the colorimetric values observed in almonds peeled with liquid nitrogen suggest that this method may better preserve the natural color of almond kernels by avoiding thermal stress associated with conventional blanching.

## 4. Conclusions

This study demonstrated that almond skins represent a valuable source of polyphenols, whose recovery is strongly influenced by both the peeling process and the cultivar. The screening of a germplasm collection highlighted a pronounced variability in total polyphenol content, antioxidant activity, and skin yield among the 106 almond cultivars, confirming the key role of genotype in determining the functional potential of this by-product.

Conventional hot-water peeling resulted in a significant loss of bioactive compounds into the blanching water, reducing the amount of polyphenols retained in the solid fraction. In contrast, liquid nitrogen peeling showed potential as an alternative approach to enhance the preservation of polyphenols and antioxidant activity in almond skins, while maintaining kernel quality, as confirmed by color analysis.

The application of a multivariate approach (PCA) allowed the identification of cultivars best suited for the recovery of bioactive-rich skins, emphasizing the importance of combining cultivar selection with processing strategies.

Overall, these findings suggest that cryogenic peeling may represent a promising tool for the valorization of almond skins as functional by-products, supporting their reuse as ingredients in food and nutraceutical applications. Further studies will be necessary to optimize process parameters and to evaluate the scalability and practical feasibility of the proposed approach.

Although the evaluation of the cost-effectiveness of liquid nitrogen peeling was beyond the scope of the present study, potential advantages such as the absence of water use and the elimination of the drying step suggest that this approach may represent a promising alternative to conventional blanching. Future studies should include additional antioxidant assays based on different reaction mechanisms (e.g., FRAP or ABTS) to provide a more comprehensive characterization of the antioxidant capacity, as well as dedicated techno-economic assessments to fully evaluate the industrial applicability of liquid nitrogen peeling.

## Figures and Tables

**Table 1:** Skin yield (%) of the examined almond cultivars, expressed as the percentage of tegument weight relative to whole kernel weight.

Cultivar	Skin Yield (%)	Cultivar	Skin Yield (%)	Cultivar	Skin Yield (%)
Don Pitrino	8.78 ± 0.13	Montagna	6.90 ± 0.06	Acquaviva	6.14 ± 0.07
Enna 2	8.73 ± 0.10	Cavaliere	6.89 ± 0.07	Filippo Ceo	6.14 ± 0.02
Bennici	8.67 ± 0.06	Milocca	6.83 ± 0.16	Carrubina	6.13 ± 0.19
Miuzza	8.60 ± 0.04	Cavalera (Bronte)	6.79 ± 0.11	Filippazzo	6.13 ± 0.02
Buscarina	8.58 ± 0.02	Lisciannarisa	6.78 ± 0.15	Falsa Pizzuta	6.11 ± 0.03
Zagarri’	8.15 ± 0.08	Sarbaggia Di Patito	6.75 ± 0.10	Fellamasa Casteltermini	6.08 ± 0.18
Nivera Manza	8.05 ± 0.10	Scummissa	6.75 ± 0.11	Staccia	6.06 ± 0.09
Fastuchina	8.03 ± 0.18	Bari Rachela (Licata)	6.70 ± 0.10	Bari Sabittisa	6.04 ± 0.07
Bronte 1	7.96 ± 0.10	Piatta Mollisa	6.70 ± 0.10	Persichina	6.04 ± 0.09
Marra’ (Mennula Di)	7.96 ± 0.10	Giardinella	6.69 ± 0.13	Chiricupara	6.00 ± 0.10
Griddetta	7.92 ± 0.12	Bargellera	6.68 ± 0.12	Cesaro’ 1	5.94 ± 0.09
Romana (Ispica)	7.89 ± 0.12	Mastraciccia	6.68 ± 0.06	Laurenne	5.93 ± 0.02
Mannara Di Chianu	7.82 ± 0.10	Regina	6.67 ± 0.12	Mennula Du Nigliu	5.92 ± 0.08
Gaglio	7.70 ± 0.07	Pilusedda	6.64 ± 0.07	Mullisa Piccola	5.84 ± 0.17
Selvatica Favata	7.64 ± 0.12	Belvedere	6.61 ± 0.04	Zottafunnuta	5.81 ± 0.08
Romana (Licata)	7.51 ± 0.10	Bari Flores	6.57 ± 0.06	Lumia	5.78 ± 0.14
Zaccaneddara	7.49 ± 0.10	Nerone	6.54 ± 0.07	Mennula Du Vattiu	5.77 ± 0.09
Mirabile	7.41 ± 0.11	Tabacchina	6.53 ± 0.09	Bonamuruni	5.68 ± 0.10
Callara	7.40 ± 0.01	Ferraduel	6.51 ± 0.05	Firruzza	5.65 ± 0.18
Nuciddara	7.39 ± 0.03	Calamonaci	6.49 ± 0.02	Zarbara	5.63 ± 0.15
Mezzalira	7.38 ± 0.11	Reginella	6.46 ± 0.00	Baggiana	5.59 ± 0.12
Cacinova	7.31 ± 0.07	Di Giorgio	6.45 ± 0.09	Cuti	5.55 ± 0.03
Tunnulidda (Soat Licata)	7.28 ± 0.10	Bottara	6.43 ± 0.14	Palma	5.47 ± 0.07
Ferrara	7.24 ± 0.20	Comunista	6.42 ± 0.06	Tuono	5.46 ± 0.07
Genco	7.18 ± 0.10	Tricula	6.41 ± 0.10	Supernova	5.45 ± 0.02
Uova Di Cucco	7.17 ± 0.08	Chiatta	6.39 ± 0.08	Amara Di Martorana	5.38 ± 0.07
Nambaredda	7.15 ± 0.04	Favaro (Lercara F.)	6.39 ± 0.03	Vaiana (Mennula Di)	5.35 ± 0.14
Perciavisazza	7.13 ± 0.08	Ferragnes	6.37 ± 0.11	Bianculidda Di Pezzino	5.33 ± 0.11
Pizzutella	7.12 ± 0.07	Universo	6.34 ± 0.15	Texas	5.27 ± 0.12
Zammuto 2	7.10 ± 0.10	Sancisuca (Soat Licata)	6.32 ± 0.10	Cavalera (Aragona)	5.14 ± 0.07
Mullisa Grande	7.00 ± 0.05	Sarbaggia Di Sciascia	6.30 ± 0.14	Pizzuta Contino	5.11 ± 0.13
Naro 1	7.00 ± 0.08	Cumma	6.26 ± 0.10	Angelica	5.07 ± 0.09
Falsa Pizzuta 2	6.97 ± 0.10	Pullara (Di Fauma)	6.22 ± 0.10	Fascionello	4.87 ± 0.07
Barunissa	6.96 ± 0.10	Cacciatura	6.16 ± 0.04	Muddisa Tonda	4.83 ± 0.03
Cuore	6.96 ± 0.04	Chiarchiara	6.15 ± 0.10	Cavalera Di Naro	4.74 ± 0.10
Buscemi	6.94 ± 0.09				

Results are expressed as mean ± standard deviation.

**Table 2:** Polyphenol content in TBP fractions and Total Recoverable Polyphenols (TRPs) of almond cultivars.

Cultivar	Total Polyphenol Content		Total Recoverable Polyphenols (TRPs)	Class *
	TPB-Water	TBP-Skin		
Pizzutella	834.49 ± 31.84	0.00 ± 0.00	834.49 ± 31.84	High
Zaccaneddara	655.68 ± 92.91	25.64 ± 8.37	681.33 ± 84.54	
Barunissa	577.52 ± 27.55	0.00 ± 0.00	577.52 ± 27.55	
Universo	517.48 ± 76.59	53.10 ± 1.78	570.58 ± 78.37	
Supernova	554.77 ± 72.40	0.00 ± 0.00	554.77 ± 72.40	
Pizzuta Contino	529.23 ± 26.26	0.00 ± 0.00	529.23 ± 26.26	
Tuono	389.15 ± 31.49	86.33 ± 12.21	475.48 ± 19.27	
Chiarchiara	429.28 ± 51.42	31.22 ± 10.77	460.49 ± 40.65	
Cavaliere	422.05 ± 56.06	16.69 ± 1.08	438.74 ± 57.14	
Carrubina	397.79 ± 37.74	21.68 ± 3.87	419.48 ± 41.61	
Laurenne	396.70 ± 22.32	17.14 ± 3.22	413.84 ± 25.53	
Pullara (Di Fauma)	396.47 ± 15.98	15.81 ± 2.23	412.28 ± 18.21	
Ferrara	393.94 ± 26.73	16.19 ± 2.06	410.13 ± 24.67	
Vaiana (Mennula Di)	359.54 ± 19.03	39.99 ± 12.47	399.52 ± 6.56	Medium
Muddisa Tonda	399.02 ± 18.38	0.00 ± 0.00	399.02 ± 18.38	
Sarbaggia Di Patito	382.12 ± 18.06	0.00 ± 0.00	382.12 ± 18.06	
Bari Rachela (Licata)	325.43 ± 10.23	55.43 ± 9.61	380.86 ± 0.62	
Naro 1	340.94 ± 31.69	38.19 ± 7.63	379.13 ± 24.06	
Genco	376.94 ± 33.72	0.00 ± 0.00	376.94 ± 33.72	
Favaro (Lercara F.)	346.49 ± 7.84	19.33 ± 0.80	365.82 ± 7.04	
Cuore	350.01 ± 20.80	0.00 ± 0.00	350.01 ± 20.80	
Fascionello	243.60 ± 30.50	105.50 ± 5.94	349.10 ± 24.56	
Ferragnes	333.62 ± 50.85	12.96 ± 1.22	346.58 ± 52.06	
Nuciddara	343.74 ± 22.90	0.00 ± 0.00	343.74 ± 22.90	
Zagarri’	331.65 ± 23.02	10.55 ± 0.97	342.21 ± 23.99	
Persichina	317.21 ± 8.44	18.58 ± 0.79	335.79 ± 9.23	
Belvedere	301.69 ± 26.52	24.87 ± 3.11	326.56 ± 29.64	
Romana (Ispica)	276.18 ± 27.05	40.41 ± 10.10	316.60 ± 16.95	
Bari Flores	302.91 ± 21.80	12.92 ± 3.44	315.82 ± 25.24	
Falsa Pizzuta 2	300.25 ± 4.39	9.31 ± 1.76	309.57 ± 6.15	
Bari Sabittisa	306.00 ± 17.54	0.00 ± 0.00	306.00 ± 17.54	
Gaglio	305.49 ± 15.24	0.00 ± 0.00	305.49 ± 15.24	
Filippo Ceo	304.22 ± 3.73	0.00 ± 0.00	304.22 ± 3.73	
Reginella	297.23 ± 5.78	0.00 ± 0.00	297.23 ± 5.78	
Romana (Licata)	282.47 ± 13.00	14.21 ± 0.63	296.69 ± 13.64	
Lumia	268.10 ± 16.36	27.35 ± 6.85	295.45 ± 9.51	
Marra’ (Mennula Di)	281.07 ± 29.70	14.19 ± 1.61	295.26 ± 31.31	
Firruzza	292.08 ± 11.55	0.00 ± 0.00	292.08 ± 11.55	
Fastuchina	263.43 ± 41.97	24.35 ± 7.57	287.79 ± 34.40	
Falsa Pizzuta	269.94 ± 26.86	12.90 ± 2.55	282.85 ± 29.40	
Ferraduel	278.26 ± 30.67	0.00 ± 0.00	278.26 ± 30.67	
Bronte 1	276.89 ± 46.99	0.00 ± 0.00	276.89 ± 46.99	
Milocca	272.73 ± 13.14	0.00 ± 0.00	272.73 ± 13.14	
Montagna	246.74 ± 14.02	17.04 ± 3.00	263.78 ± 17.02	
Don Pitrino	262.97 ± 20.41	0.00 ± 0.00	262.97 ± 20.41	
Bianculidda Di Pezzino	259.40 ± 15.99	0.00 ± 0.00	259.40 ± 15.99	
Miuzza	256.87 ± 21.61	0.00 ± 0.00	256.87 ± 21.61	
Angelica	255.37 ± 21.33	0.00 ± 0.00	255.37 ± 21.33	
Comunista	254.01 ± 4.71	0.00 ± 0.00	254.01 ± 4.71	
Cesaro’ 1	253.77 ± 11.50	0.00 ± 0.00	253.77 ± 11.50	
Cacciatura	189.24 ± 13.20	62.34 ± 10.13	251.58 ± 3.07	
Buscarina	250.96 ± 8.82	0.00 ± 0.00	250.96 ± 8.82	
Baggiana	210.50 ± 29.54	35.93 ± 6.49	246.43 ± 36.03	
Cavalera Di Naro	245.03 ± 6.36	0.00 ± 0.00	245.03 ± 6.36	
Giardinella	231.16 ± 5.56	10.20 ± 0.48	241.36 ± 6.04	
Sarbaggia Di Sciascia	225.70 ± 20.91	12.97 ± 2.62	238.68 ± 23.52	
Chiricupara	238.09 ± 2.63	0.00 ± 0.00	238.09 ± 2.63	
Uova Di Cucco	217.61 ± 13.24	13.63 ± 1.71	231.24 ± 14.95	
Callara	204.83 ± 11.75	25.43 ± 5.07	230.26 ± 16.82	
Mannara Di Chianu	209.98 ± 60.53	19.52 ± 0.30	229.50 ± 60.84	
Cuti	226.59 ± 33.63	0.00 ± 0.00	226.59 ± 33.63	
Cacinova	225.21 ± 18.28	0.00 ± 0.00	225.21 ± 18.28	
Nivera Manza	207.62 ± 21.50	17.12 ± 1.61	224.74 ± 19.89	
Perciavisazza	222.25 ± 1.63	0.00 ± 0.00	222.25 ± 1.63	
Pilusedda	191.21 ± 3.53	29.34 ± 3.39	220.56 ± 6.93	
Di Giorgio	216.10 ± 12.58	0.00 ± 0.00	216.10 ± 12.58	
Cavalera (Bronte)	212.25 ± 12.37	0.00 ± 0.00	212.25 ± 12.37	
Amara Di Martorana	208.85 ± 10.48	0.00 ± 0.00	208.85 ± 10.48	
Chiatta	208.01 ± 11.78	0.00 ± 0.00	208.01 ± 11.78	
Mirabile	204.46 ± 10.79	0.00 ± 0.00	204.46 ± 10.79	
Acquaviva	168.98 ± 10.28	34.14 ± 3.68	203.12 ± 13.95	
Bennici	192.17 ± 4.97	10.85 ± 2.48	203.02 ± 7.45	
Mennula Du Vattiu	170.30 ± 24.67	32.51 ± 1.75	202.81 ± 22.92	
Cumma	201.92 ± 3.21	0.00 ± 0.00	201.92 ± 3.21	
Tabacchina	199.33 ± 10.20	0.00 ± 0.00	199.33 ± 10.20	Low
Mullisa Grande	191.70 ± 4.24	0.00 ± 0.00	191.70 ± 4.24	
Selvatica Favata	189.94 ± 1.87	0.00 ± 0.00	189.94 ± 1.87	
Zottafunnuta	174.12 ± 3.11	12.69 ± 1.00	186.81 ± 4.11	
Nambaredda	185.08 ± 1.35	0.00 ± 0.00	185.08 ± 1.35	
Texas	155.42 ± 13.56	26.65 ± 2.19	182.07 ± 15.76	
Staccia	181.29 ± 1.22	0.00 ± 0.00	181.29 ± 1.22	
Zarbara	179.72 ± 23.97	0.00 ± 0.00	179.72 ± 23.97	
Sancisuca (Soat Licata)	152.67 ± 0.38	20.94 ± 0.88	173.60 ± 0.51	
Mennula Du Nigliu	168.66 ± 7.74	0.00 ± 0.00	168.66 ± 7.74	
Enna 2	165.77 ± 5.69	0.00 ± 0.00	165.77 ± 5.69	
Bargellera	153.46 ± 19.44	12.17 ± 1.54	165.64 ± 20.98	
Buscemi	164.25 ± 7.84	0.00 ± 0.00	164.25 ± 7.84	
Piatta Mollisa	159.21 ± 0.89	0.00 ± 0.00	159.21 ± 0.89	
Mullisa Piccola	157.10 ± 12.25	0.00 ± 0.00	157.10 ± 12.25	
Cavalera (Aragona)	118.14 ± 6.68	29.54 ± 3.28	147.68 ± 9.96	
Griddetta	145.76 ± 28.30	0.00 ± 0.00	145.76 ± 28.30	
Calamonaci	143.59 ± 0.63	0.00 ± 0.00	143.59 ± 0.63	
Regina	127.08 ± 3.49	13.81 ± 0.77	140.89 ± 4.26	
Tunnulidda (Soat Licata)	136.23 ± 15.25	0.00 ± 0.00	136.23 ± 15.25	
Fellamasa Casteltermini	113.83 ± 2.04	15.83 ± 1.30	129.65 ± 0.74	
Bottara	129.27 ± 2.41	0.00 ± 0.00	129.27 ± 2.41	
Zammuto 2	122.41 ± 10.95	0.00 ± 0.00	122.41 ± 10.95	
Mastraciccia	108.55 ± 5.96	9.81 ± 0.35	118.36 ± 5.61	
Mezzalira	115.60 ± 11.34	0.00 ± 0.00	115.60 ± 11.34	
Nerone	81.63 ± 0.54	28.01 ± 4.98	109.64 ± 5.52	
Bonamuruni	103.51 ± 12.59	0.00 ± 0.00	103.51 ± 12.59	
Filippazzo	101.86 ± 9.04	0.00 ± 0.00	101.86 ± 9.04	
Tricula	99.02 ± 2.58	0.00 ± 0.00	99.02 ± 2.58	
Scummissa	96.68 ± 5.03	0.00 ± 0.00	96.68 ± 5.03	
Lisciannarisa	89.07 ± 2.90	0.00 ± 0.00	89.07 ± 2.90	
Palma	52.77 ± 3.54	0.00 ± 0.00	52.77 ± 3.54	

Results are expressed as mg GAE/100 g of almonds (mean ± standard deviation). * Classification based on TRP: High (>400 mg GAE/100 g), Medium (200–400 mg GAE/100 g), Low (<200 mg GAE/100 g).

**Table 3:** Antioxidant activity in TBP fractions and Total Recoverable Antioxidant Activity (TRAA) of almond cultivars.

Cultivar	Antioxidant Activity		Total Recoverable Antioxidant Activity (TRAA)	Class
	TBP-Water	TBP-Skin		
Nivera Manza	3051.98 ± 142.74	78.27 ± 3.36	3130.25 ± 143.51	High
Reginella	3054.02 ± 166.13	27.25 ± 1.37	3081.27 ± 166.70	
Milocca	3022.93 ± 111.02	21.13 ± 0.56	3044.06 ± 111.52	
Barunissa	2999.32 ± 108.27	39.62 ± 2.56	3038.94 ± 106.97	
Mullisa Grande	3016.42 ± 122.57	17.16 ± 0.41	3033.58 ± 122.84	
Favaro (Lercara F.)	2845.41 ± 86.68	101.46 ± 8.48	2946.87 ± 91.52	
Enna 2	2877.48 ± 139.39	25.45 ± 1.33	2902.93 ± 138.26	
Cavaliere	2825.40 ± 213.40	75.83 ± 3.76	2901.23 ± 214.42	
Palma	2647.88 ± 113.17	0.00 ± 0.00	2647.88 ± 113.17	
Tabacchina	2537.41 ± 162.54	21.70 ± 1.23	2559.11 ± 163.39	
Zottafunnuta	2500.00 ± 23.32	36.38 ± 1.64	2536.38 ± 23.93	
Tuono	2287.99 ± 147.82	185.92 ± 9.42	2473.91 ± 152.55	Medium
Vaiana (Mennula Di)	2264.09 ± 69.31	139.89 ± 3.19	2403.98 ± 70.01	
Cacciatura	2214.15 ± 87.93	181.57 ± 11.33	2395.72 ± 98.68	
Bari Rachela (Licata)	2228.14 ± 217.14	160.84 ± 4.72	2388.98 ± 214.27	
Chiarchiara	2260.01 ± 190.13	126.55 ± 8.91	2386.56 ± 181.46	
Naro 1	2270.89 ± 80.22	97.94 ± 5.82	2368.83 ± 82.42	
Tricula	2353.87 ± 103.17	8.89 ± 0.23	2362.76 ± 103.00	
Falsa Pizzuta 2	2308.98 ± 59.78	39.30 ± 1.92	2348.28 ± 61.57	
Zagarri’	2299.65 ± 133.92	42.70 ± 3.10	2342.35 ± 130.94	
Buscemi	2327.44 ± 107.98	3.10 ± 0.29	2330.54 ± 108.22	
Marra’ (Mennula Di)	2264.28 ± 147.64	63.22 ± 1.55	2327.50 ± 147.27	
Cumma	2273.61 ± 222.89	44.94 ± 2.37	2318.55 ± 221.09	
Tunnulidda (Soat Licata)	2302.18 ± 120.30	10.76 ± 0.48	2312.94 ± 120.40	
Regina	2269.82 ± 110.56	37.24 ± 2.46	2307.06 ± 108.90	
Mastraciccia	2253.30 ± 37.23	47.83 ± 2.84	2301.13 ± 39.96	
Cavalera (Aragona)	2208.51 ± 24.37	85.53 ± 4.15	2294.04 ± 22.74	
Montagna	2248.35 ± 28.28	43.09 ± 2.71	2291.44 ± 26.61	
Bari Sabittisa	2257.87 ± 94.91	33.52 ± 0.23	2291.39 ± 95.05	
Cavalera Di Naro	2281.19 ± 120.37	2.70 ± 0.04	2283.89 ± 120.35	
Texas	2197.63 ± 54.85	70.84 ± 3.84	2268.47 ± 55.05	
Carrubina	2208.90 ± 24.58	52.95 ± 4.51	2261.85 ± 20.85	
Universo	2052.66 ± 62.15	205.75 ± 12.30	2258.41 ± 66.49	
Cavalera (Bronte)	2218.81 ± 169.36	32.39 ± 1.21	2251.20 ± 168.70	
Di Giorgio	2231.44 ± 39.81	13.03 ± 0.56	2244.47 ± 39.26	
Belvedere	2095.61 ± 108.73	137.51 ± 2.29	2233.12 ± 110.93	
Ferraduel	2199.77 ± 103.38	33.30 ± 1.65	2233.07 ± 101.96	
Persichina	2194.71 ± 171.27	35.99 ± 1.98	2230.70 ± 171.91	
Uova Di Cucco	2071.32 ± 161.51	110.90 ± 6.65	2182.22 ± 168.14	
Pilusedda	2060.24 ± 100.02	102.78 ± 6.01	2163.02 ± 95.05	
Acquaviva	2010.69 ± 113.21	141.61 ± 14.19	2152.30 ± 125.52	
Baggiana	2004.08 ± 82.19	146.32 ± 7.72	2150.40 ± 74.63	
Fastuchina	2038.28 ± 56.02	100.02 ± 5.39	2138.30 ± 60.57	
Mennula Du Vattiu	2002.91 ± 61.00	107.02 ± 3.25	2109.93 ± 58.72	
Ferragnes	2011.85 ± 60.26	83.95 ± 3.97	2095.80 ± 58.96	
Comunista	2025.07 ± 95.46	66.70 ± 3.46	2091.77 ± 94.08	
Genco	2029.34 ± 147.62	57.10 ± 2.14	2086.44 ± 149.38	
Chiricupara	2017.49 ± 85.26	65.62 ± 2.23	2083.11 ± 83.06	
Bronte 1	2059.08 ± 126.38	21.53 ± 1.38	2080.61 ± 125.08	
Romana (Ispica)	1894.68 ± 100.69	178.82 ± 5.40	2073.50 ± 105.15	
Zarbara	1987.37 ± 70.70	85.79 ± 1.59	2073.16 ± 69.16	
Zaccaneddara	1982.90 ± 57.27	88.39 ± 2.70	2071.29 ± 54.57	
Supernova	2033.81 ± 67.35	35.94 ± 2.77	2069.75 ± 68.46	
Miuzza	2032.06 ± 111.96	33.99 ± 0.51	2066.05 ± 112.42	
Cuore	2012.24 ± 79.89	51.50 ± 1.37	2063.74 ± 79.59	
Bianculidda Di Pezzino	1950.25 ± 176.18	111.97 ± 2.74	2062.22 ± 178.66	
Gaglio	2015.35 ± 82.41	45.12 ± 3.53	2060.47 ± 78.89	
Sancisuca (Soat Licata)	1973.77 ± 166.25	86.33 ± 6.61	2060.10 ± 159.68	
Griddetta	2029.15 ± 32.57	30.75 ± 1.72	2059.90 ± 32.12	
Ferrara	2006.41 ± 150.57	52.72 ± 1.96	2059.13 ± 149.82	
Nambaredda	2021.18 ± 88.06	32.41 ± 2.18	2053.59 ± 87.20	
Cesaro’ 1	2031.29 ± 155.22	16.91 ± 1.31	2048.20 ± 153.92	
Mannara Di Chianu	1959.58 ± 75.04	71.72 ± 4.93	2031.30 ± 78.47	
Nuciddara	2020.60 ± 85.89	2.84 ± 0.17	2023.44 ± 85.91	
Bargellera	1959.58 ± 50.63	59.92 ± 0.82	2019.50 ± 50.75	
Amara Di Martorana	2014.96 ± 97.81	2.96 ± 0.09	2017.92 ± 97.84	
Scummissa	2011.85 ± 93.34	2.66 ± 0.13	2014.51 ± 93.36	
Mennula Du Nigliu	1982.71 ± 108.84	31.23 ± 1.07	2013.94 ± 107.81	
Buscarina	1989.70 ± 48.41	21.16 ± 0.73	2010.86 ± 49.11	
Staccia	1999.03 ± 83.53	8.60 ± 0.19	2007.63 ± 83.37	
Giardinella	1957.05 ± 166.25	50.01 ± 1.26	2007.06 ± 167.16	
Mullisa Piccola	1974.15 ± 141.47	31.62 ± 2.26	2005.77 ± 139.23	
Sarbaggia Di Sciascia	1943.84 ± 88.96	59.63 ± 1.88	2003.47 ± 87.48	
Perciavisazza	1993.59 ± 121.96	8.28 ± 0.25	2001.87 ± 121.71	
Piatta Mollisa	1974.93 ± 39.22	22.88 ± 1.36	1997.81 ± 39.83	Low
Nerone	1921.30 ± 29.11	65.54 ± 3.00	1986.84 ± 26.19	
Lisciannarisa	1979.21 ± 67.78	5.52 ± 0.20	1984.73 ± 67.84	
Fellamasa Casteltermini	1945.39 ± 71.86	39.32 ± 1.63	1984.71 ± 71.74	
Falsa Pizzuta	1923.24 ± 35.46	56.93 ± 2.49	1980.17 ± 33.65	
Firruzza	1949.48 ± 98.16	28.81 ± 1.83	1978.29 ± 96.75	
Mirabile	1965.80 ± 48.97	12.36 ± 0.77	1978.16 ± 48.55	
Bari Flores	1917.02 ± 79.99	55.81 ± 2.50	1972.83 ± 82.44	
Fascionello	1834.82 ± 72.99	134.64 ± 6.65	1969.46 ± 79.53	
Romana (Licata)	1902.06 ± 55.68	55.84 ± 2.11	1957.90 ± 53.78	
Bonamuruni	1900.31 ± 98.54	29.52 ± 1.05	1929.83 ± 98.22	
Bennici	1878.35 ± 59.76	38.45 ± 1.06	1916.80 ± 60.34	
Selvatica Favata	1900.89 ± 105.52	8.60 ± 0.39	1909.49 ± 105.77	
Lumia	1794.21 ± 130.46	108.62 ± 6.62	1902.83 ± 123.95	
Don Pitrino	1876.80 ± 70.81	1.81 ± 0.08	1878.61 ± 70.82	
Filippo Ceo	1877.38 ± 70.09	0.00 ± 0.00	1877.38 ± 70.09	
Filippazzo	1865.53 ± 36.67	9.61 ± 0.36	1875.14 ± 36.32	
Pizzutella	1829.97 ± 73.51	40.44 ± 1.79	1870.41 ± 71.74	
Callara	1763.70 ± 92.09	100.66 ± 4.70	1864.36 ± 95.11	
Sarbaggia Di Patito	1844.35 ± 70.55	8.91 ± 0.37	1853.26 ± 70.86	
Pizzuta Contino	1849.20 ± 31.67	1.10 ± 0.04	1850.30 ± 31.67	
Pullara (Di Fauma)	1797.12 ± 91.13	51.71 ± 4.34	1848.83 ± 88.72	
Muddisa Tonda	1831.52 ± 74.48	10.89 ± 0.32	1842.41 ± 74.78	
Laurenne	1804.90 ± 101.47	32.31 ± 2.49	1837.21 ± 103.41	
Angelica	1810.14 ± 82.35	0.00 ± 0.00	1810.14 ± 82.35	
Cacinova	1803.73 ± 72.40	3.72 ± 0.29	1807.45 ± 72.48	
Cuti	1730.86 ± 59.31	8.25 ± 0.18	1739.11 ± 59.32	
Zammuto 2	1719.98 ± 69.69	14.53 ± 0.91	1734.51 ± 69.57	
Mezzalira	1705.40 ± 116.55	8.05 ± 0.33	1713.45 ± 116.22	
Bottara	1663.82 ± 29.28	17.25 ± 0.52	1681.07 ± 29.49	
Chiatta	1636.61 ± 59.27	16.31 ± 0.64	1652.92 ± 58.90	
Calamonaci	1651.77 ± 82.07	0.00 ± 0.00	1651.77 ± 82.07	

Results are expressed as µg TE/100 g of almonds (mean ± standard deviation).

**Table 4:** Distribution of selected almond cultivars across PCA quadrants and their interpretation based on total bioactive content (TRP, TRAA) and skin yield (%).

PCA Quadrant	Interpretation	Selected Cultivars
I (+PC1, +PC2)	High antioxidant activity, high polyphenol content, and favorable skin yield	Universo; Tuono; Chiarchiara; Naro 1; Bari Rachela (Licata); Cavaliere; Lumia; Barunissa; Regina
II (−PC1, +PC2)	Relatively high polyphenol content and skin yield with lower antioxidant activity	Fascionello; Acquaviva; Genco; Ferragnes
III (−PC1, −PC2)	Lower bioactive content, included as reference/intermediate cultivars	Nerone; Tricula
IV (+PC1, −PC2)	Higher antioxidant activity and/or skin yield with moderate polyphenol content	Romana (Licata); Don Pitrino; Buscemi; Enna 2; Bennici

**Table 5:** CIELab* color parameters (L*, a*, b*, C*, and h°) of almond kernels obtained by Traditional Blanching Peeling (TBP) and liquid nitrogen peeling (LNP).

Cultivar	Treatment	L*	a*	b*	C*	h°
Barunissa	TBP	71.20 ± 3.36	2.21 ± 1.37	15.45 ± 1.09	15.65 ± 1.21	82.04 ± 4.57
	LNP	82.66 ± 2.43	4.03 ± 0.62	16.82 ± 1.61	17.31 ± 1.55	76.41 ± 2.55
Buscemi	TBP	74.82 ± 1.81	1.72 ± 0.18	14.42 ± 1.05	14.53 ± 1.06	83.22 ± 0.34
	LNP	80.39 ± 3.74	4.64 ± 0.92	18.82 ± 2.12	19.39 ± 2.21	76.20 ± 2.06
Cavaliere	TBP	73.06 ± 3.91	1.47 ± 0.34	15.25 ± 1.52	15.33 ± 1.48	84.37 ± 1.82
	LNP	79.86 ± 1.75	5.23 ± 0.71	17.07 ± 1.31	17.88 ± 1.09	72.83 ± 3.28
Regina	TBP	77.55 ± 2.56	0.96 ± 0.83	15.53 ± 1.30	15.58 ± 1.29	86.48 ± 3.28
	LNP	81.98 ± 3.29	4.64 ± 0.66	17.74 ± 1.33	18.35 ± 1.19	75.25 ± 2.78
Enna 2	TBP	72.40 ± 1.96	1.77 ± 0.46	15.12 ± 0.95	15.22 ± 0.98	83.35 ± 1.53
	LNP	78.43 ± 2.44	5.98 ± 1.04	20.64 ± 1.82	21.50 ± 1.98	73.91 ± 1.85
Don Pitrinu	TBP	71.70 ± 2.56	1.98 ± 0.88	16.57 ± 1.03	16.70 ± 1.12	83.32 ± 2.49
	LNP	77.78 ± 4.64	5.28 ± 0.95	17.55 ± 1.59	18.35 ± 1.55	73.21 ± 3.14
Universo	TBP	79.64 ± 0.52	1.89 ± 0.43	13.78 ± 0.29	13.92 ± 0.32	82.22 ± 1.65
	LNP	78.17 ± 1.46	4.13 ± 0.75	18.28 ± 2.04	18.76 ± 1.92	77.08 ± 2.99
Genco	TBP	75.67 ± 1.47	3.28 ± 0.32	14.55 ± 1.56	14.92 ± 1.47	77.14 ± 2.25
	LNP	71.74 ± 2.60	7.35 ± 0.39	17.78 ± 1.66	19.25 ± 1.56	67.41 ± 2.08
Bennici	TBP	76.88 ± 2.55	2.04 ± 0.78	13.01 ± 1.39	13.20 ± 1.32	80.84 ± 3.69
	LNP	80.81 ± 1.98	6.18 ± 0.78	14.85 ± 1.30	16.09 ± 1.34	67.37 ± 2.55
Ferragnes	TBP	74.94 ± 3.30	7.60 ± 1.35	22.34 ± 2.74	23.61 ± 2.97	71.26 ± 1.81
	LNP	76.62 ± 2.61	6.03 ± 1.35	17.21 ± 1.64	18.28 ± 1.53	70.61 ± 4.51
Acquaviva	TBP	78.34 ± 3.50	5.09 ± 1.19	21.89 ± 3.08	22.54 ± 2.75	76.46 ± 4.70
	LNP	77.08 ± 2.68	4.28 ± 0.80	18.32 ± 3.24	18.85 ± 3.05	76.38 ± 4.37
Chiarchiara	TBP	78.38 ± 4.49	7.21 ± 0.83	21.68 ± 2.59	22.85 ± 2.70	71.59 ± 0.81
	LNP	83.35 ± 2.92	4.08 ± 0.99	19.63 ± 3.20	20.09 ± 2.97	77.93 ± 3.74
Tricula	TBP	81.42 ± 3.06	5.17 ± 0.96	21.97 ± 2.05	22.60 ± 1.91	77.78 ± 1.51
	LNP	78.94 ± 2.31	4.87 ± 0.68	16.61 ± 0.67	17.32 ± 0.80	73.71 ± 1.72
Nerone	TBP	80.46 ± 2.36	3.58 ± 1.52	21.03 ± 0.84	21.37 ± 0.85	81.84 ± 2.08
	LNP	78.52 ± 1.31	4.89 ± 0.56	19.19 ± 1.38	19.82 ± 1.27	75.61 ± 2.26
Naro 1	TBP	79.21 ± 2.92	4.79 ± 1.28	18.54 ± 1.61	19.19 ± 1.43	75.36 ± 4.44
	LNP	78.56 ± 2.67	5.13 ± 1.34	19.42 ± 0.87	20.12 ± 0.86	75.21 ± 3.82
Tuono	TBP	79.56 ± 1.51	4.44 ± 0.44	15.69 ± 1.15	16.32 ± 1.06	74.12 ± 2.22
	LNP	81.60 ± 2.73	4.73 ± 0.79	19.30 ± 2.08	19.89 ± 1.97	76.07 ± 2.82
Bari Rachela (Licata)	TBP	82.91 ± 3.63	4.08 ± 1.10	17.60 ± 1.96	18.11 ± 1.83	76.79 ± 4.43
	LNP	80.93 ± 3.12	3.95 ± 1.44	16.70 ± 2.35	17.24 ± 2.04	76.15 ± 6.02
Fascionello	TBP	78.43 ± 4.35	7.08 ± 0.98	20.54 ± 1.55	21.74 ± 1.58	70.98 ± 2.37
	LNP	78.90 ± 2.47	6.21 ± 1.39	15.59 ± 0.56	16.83 ± 0.56	68.32 ± 4.74
Lumia	TBP	76.74 ± 5.27	6.51 ± 1.32	17.90 ± 1.81	19.09 ± 1.73	69.95 ± 4.31
	LNP	78.51 ± 1.74	4.59 ± 0.24	17.95 ± 1.26	18.53 ± 1.26	75.64 ± 0.83
Romana (Licata)	TBP	75.25 ± 2.06	5.71 ± 0.81	24.24 ± 2.77	24.92 ± 2.73	76.64 ± 2.09
	LNP	77.63 ± 3.49	4.58 ± 1.08	15.85 ± 2.73	16.57 ± 2.42	73.32 ± 5.78

Results are expressed as mean ± standard deviation. L*, a*, and b* are the CIELab color coordinates, where L* represents lightness, a* indicates redness, and b* indicates yellowness. Chroma (C*) describes color saturation, while the hue angle (h°) represents color tone. Data for individual cultivars are descriptive. Statistical comparisons between TBP and LNP were performed on the overall dataset for each color parameter, as described in Section 2.4.

## Data Availability

The original contributions presented in this study are included in the article/Supplementary Material. Further inquiries can be directed to the corresponding author.

## Supplementary Materials

The following supporting information can be downloaded at: https://www.mdpi.com/article/10.3390/foods15101668/s1, Figure S1: Extraction efficiency of the solvent systems tested during the optimization phase.
