# Supplementary material for: Screening of Almond Germplasm for Bioactive-Rich Skin Recovery and Application of Liquid Nitrogen Peeling
Source: Foods. 2026 May 11;15(10):1668. doi: 10.3390/foods15101668 (PMC13205163; doi:10.3390/foods15101668)
Supplement: Supplementary file 1 [file foods-15-01668-s001.zip › foods-4252597-supplementary.pdf]

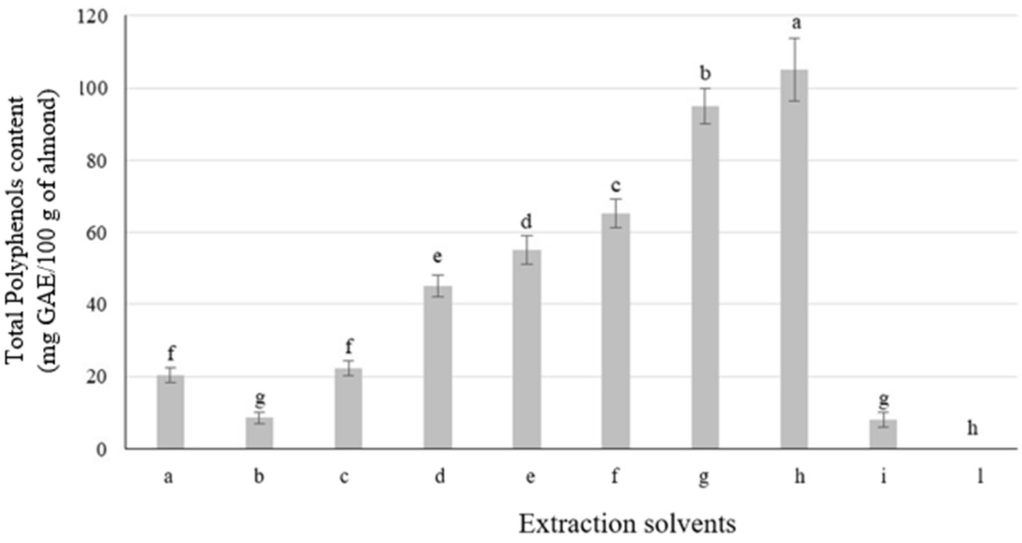

Figure S1. Extraction efficiency of the solvent systems tested during the optimization phase. Solvents are indicated as follows: (a) distilled water; (b) water acidified with 1% HCl (0.1 N); (c) water at 40 °C; (d) water at 40 °C acidified with 1% HCl (0.1 N); (e) methanol; (f) methanol acidified with 1% HCl (0.1 N); (g) ethanol; (h) ethanol acidified with 1% HCl (0.1 N); (i) isopropanol acidified with 1% HCl (0.1 N); (j) ethyl acetate. Data are expressed as mean ± standard deviation. Statistical differences among solvents were assessed by one-way ANOVA followed by Fisher's LSD test ($p < 0.05$).
